# Non-genomic thyroid hormone signaling through NO/cGMP/PKGII

**DOI:** 10.1186/2050-6511-14-S1-O26

**Published:** 2013-08-29

**Authors:** Hema Kalyanaraman, Raphaela Schwappacher, Jisha Joshua, Shunhui Zhuang, Brian Scott, Wolfgang Dillmann, John Frangos, Renate B Pilz

**Affiliations:** 1 Department of Medicine and Moores Cancer Center, University of California, San Diego, La Jolla, California 92093-0652, USA; 2 La Jolla Bioengineering Institute, La Jolla, California 92037, USA

## Background

Skeletal integrity requires continuous bone remodelling by osteoblasts and osteoclasts, and thyroid hormone (TH) is a key regulator of bone remodelling. Excess TH (hyperthyroidism) causes net bone loss, resulting in osteoporosis and increased fracture risk; lack of TH (hypothyroidism) also increases fracture risk because bones become brittle from decreased bone turnover [1]. TH stimulates bone formation and resorption through processes that are only partly defined; it enhances osteoblast proliferation and differentiation, and induces osteoblast production of the osteoclast differentiation factor RANKL (receptor activator of nuclear factor-κB ligand). Nuclear TH receptors (THR-α and THR-β) act as transcriptional regulators and generate the hormone’s classic “genomic” effects [1]. In different cell types, TH also has transcription-independent (“non-genomic”) effects, including stimulation of the MEK/Erk and PI3K/Akt/mTOR kinase cascades, but the molecular mechanisms mediating these non-genomic effects are largely unknown.

## Results

We found that physiological concentrations of 3,5,3’-triiodo-L-thyronine (T3, 10^-9^ to 10^-11^M), but not reverse-T3, rapidly increase NO production, and activate Src, Erk, and Akt in osteoblasts. These TH effects required THR-α, but were independent of THR–β. We identified a novel, membrane-bound THR-α isoform that mediates T3-induced Erk/Akt activation, but does not affect transcription from TH response element-containing promoters. Signalling via the newly-discovered THR-α isoform was blocked by inhibitors of NO synthase, guanylate cyclase (sGC), or protein kinase G (PKG), and was defective in endothelial NO synthase (eNOS)- or PKG II-deficient osteoblasts. We showed previously that NO/cGMP induce osteoblast proliferation through PKG II activation of Src and Erk, and established a mechanism for Src activation by PKG II [2]. We now show that TH enhances osteoblast proliferation and survival, and induces *osteocalcin* and *fos* family gene expression in a NO/cGMP/PKG-dependent fashion via “non-genomic” activation of Erk. In contrast, TH-induced expression of the osteoclast regulator RANKL occurs independently of NO, suggesting a classic “genomic” effect via nuclear THR-α. Consistent with these results, treatment with the sGC activator cinaciguat increased bone formation in hypothyroid mice, without affecting osteoclast numbers.

## Conclusion

We conclude that anabolic effects of TH in osteoblasts are mediated predominantly by non-genomic TH signalling, via activation of a novel, membrane-bound THR-α isoform, with subsequent activation of eNOS, sGC, PKGII, Src, Erk, and Akt. Our results are consistent with the phenotype of THR-α knockout mice and the role of NO in bone biology.
